# Selection and Validation of Reference Genes for qRT-PCR Expression Analysis of Candidate Genes Involved in Olfactory Communication in the Butterfly *Bicyclus anynana*

**DOI:** 10.1371/journal.pone.0120401

**Published:** 2015-03-20

**Authors:** Alok Arun, Véronique Baumlé, Gaël Amelot, Caroline M. Nieberding

**Affiliations:** Evolutionary Ecology and Genetics group, Biodiversity Research Centre, Earth and Life Institute, Université catholique de Louvain, Croix du Sud 4, Louvain-la-Neuve, Belgium; Ghent University, BELGIUM

## Abstract

Real-time quantitative reverse transcription PCR (qRT-PCR) is a technique widely used to quantify the transcriptional expression level of candidate genes. qRT-PCR requires the selection of one or several suitable reference genes, whose expression profiles remain stable across conditions, to normalize the qRT-PCR expression profiles of candidate genes. Although several butterfly species (Lepidoptera) have become important models in molecular evolutionary ecology, so far no study aimed at identifying reference genes for accurate data normalization for any butterfly is available. The African bush brown butterfly *Bicyclus anynana* has drawn considerable attention owing to its suitability as a model for evolutionary ecology, and we here provide a maiden extensive study to identify suitable reference gene in this species. We monitored the expression profile of twelve reference genes: *eEF-1α*, *FK506*, *UBQL40*, *RpS8*, *RpS18*, *HSP*, *GAPDH*, *VATPase*, *ACT3*, *TBP*, *eIF2* and *G6PD*. We tested the stability of their expression profiles in three different tissues (wings, brains, antennae), two developmental stages (pupal and adult) and two sexes (male and female), all of which were subjected to two food treatments (food stress and control feeding *ad libitum*). The expression stability and ranking of twelve reference genes was assessed using two algorithm-based methods, NormFinder and geNorm. Both methods identified *RpS8* as the best suitable reference gene for expression data normalization. We also showed that the use of two reference genes is sufficient to effectively normalize the qRT-PCR data under varying tissues and experimental conditions that we used in *B*. *anynana*. Finally, we tested the effect of choosing reference genes with different stability on the normalization of the transcript abundance of a candidate gene involved in olfactory communication in *B*. *anynana*, the Fatty Acyl Reductase 2, and we confirmed that using an unstable reference gene can drastically alter the expression profile of the target candidate genes.

## Introduction

Butterflies have fascinated mankind throughout the ages with their wing coloration, association with angiosperms and life cycle involving metamorphosis. These visually striking members of the class of insects are placed in the order Lepidoptera from which they diversified ~100 million years ago and show remarkable diversity with over 17,500 known species [1–3]. Over the last few decades, a significant amount of work underpinning the fields of ecology, evolution, genetics, and developmental biology has been carried out with model species of butterflies. The availability of the recently sequenced genomes of *Heliconius melpomene* [4], *Danaus plexipus* [5] and *Melitaea cinxia* [6] has offered an extraordinary opportunity to study the expression patterns of gene families in butterflies. Besides genomic resources, tissue-specific expressed sequence tags (ESTs) libraries have also been generated for certain butterflies [7,8]. The number of butterfly genome sequences available and genome projects currently underway indicate an upward trend in the interest of researchers towards this group of organisms. Yet, an extensive study aimed at identifying suitable reference genes to normalize the expression profiles of genes of interest in any butterfly species is surprisingly still lacking.

The African bush brown butterfly *Bicyclus anynana* (Nymphalidae; Satyrinae) (Butler, 1879) has emerged as a model in evolutionary developmental biology since significant advances have revealed how genetic variation between individuals interacts with developmental processes to provide adaptive phenotypic variation in wing patterns, a major trait under natural and sexual selection [9–11]. *B*. *anynana* has also become a model in ecology and evolution, thanks to the coupling of in-depth field studies and experimental work conducted on lab-adapted population. This strategy has unraveled the ecological relevance of a large range of phenotypes associated, for example, with seasonal polyphenism producing adaptive phenotypes to the alternating wet and dry seasons in East Africa, life-history traits relevant to ageing research and to sexual selection [10,12]. In this regard, it has been demonstrated that *B*. *anynana* males use a male sex pheromone (MSP hereafter), produced by wing structures named androconia, which plays a key role in mate choice and sexual selection [13–15]. After landing behind a female, *B*. *anynana* males flicker their wings and erect their androconial hair, likely favoring the dissemination of MSP at short range over the female antennae during courtship [14]. The MSP composition has been identified and includes three chemical components [14]. Variation in abundance of the three MSP components between males informs females about male phenotypic traits associated to fitness, such as age and inbreeding status, and this in turn affects male reproductive success [15]. Interestingly, the number and position of androconia is a key taxonomic trait to distinguish between closely related species of the highly speciose *Bicyclus* genus (>88 currently described species) [16,17]. Moreover, the large diversity of wing chemical composition found across a subset of 32 species of *Bicyclus* was recently shown to have contributed to the diversification of the genus by recurrent reproductive character displacement [18].

While our understanding of the adaptive value of phenotypic variation in sex pheromone communication in *B*. *anynana* is increasing, the study of its molecular basis is still in its infancy and this holds true for olfactory communication in butterflies in general. In this regard, we decided to investigate the molecular basis of sex pheromone communication in *B*. *anynana*. For this, we needed to use quantitative real-time reverse transcription PCR (qRT-PCR) to validate the variation in level of expression of candidate genes. In this respect, reliable reference genes need to be selected and validated since non-validated reference genes can drastically alter the expression profiles of genes [19]. The present study thus aims at identifying and validating suitable reference genes in *B*. *anynana* by evaluating the mRNA expression stability profile of twelve potential reference genes under two different experimental conditions employing the widely used technique of qRT-PCR. This technique has been successfully used to identify suitable reference genes in various plants, animals and insects including for example *Arabidopsis thaliana* [20], *Sus domesticus* [21]) *Drosophila melanogaster* [22], *Spodoptera exigua* [23] *Bombyx mori* [24], *Chilo suppressalis* [25], *Plutella xylostella* [26], *Helicoverpa armigera* [27], *Agrotis ipsilon* [28] and *Spodoptera litura* [29]. As previous studies have revealed that variation in the expression profiles of reference genes are often observed in varying experimental procedures, it has become mandatory to test multiple reference genes in multiple experimental conditions to select the best reference genes for normalization studies in any given tissue set [30,31] or experimental condition [32]. Moreover, the number of reference genes needed to accurately normalize the qRT-PCR expression data must be determined on a case by case basis since using one reference gene can induce errors in the results [33]. We thus used here two algorithm-based methods, NormFinder [34] and geNorm [35] for identifying suitable reference genes, to: (a) identify the best reference gene among our 12 candidates genes; and (b) ascertain the number of reference genes needed for optimizing the normalization of gene expression levels in *B*. *anynana*. We tested our 12 candidate reference genes by sampling eight tissues of insects reared under two different experimental conditions (food stress and control feeding *ad libitum*). Finally, we validated the selection of our reference genes by evaluating across different tissues, the expression profile of a fatty-acyl reductase gene (*FAR 2* hereafter) involved in sex pheromone biosynthesis in the Lepidoptera species *B*. *anynana* [36].

## Materials and Methods

### Rearing of insects

Experiments were performed with *B*. *anynana* from an outbred laboratory stock population established in the laboratory from eggs collected from a laboratory raised colony in Leiden, The Netherlands, which was itself originally established from 80 gravid females collected in Malawi in 1988 [37]. Around 200 to 400 adults per generation are reared in order to maintain high levels of heterozygosity [38]. Animals were reared in a climate room under a standard temperature regime (27°C ± 2°C), 12:12 L:D and high relative humidity (66% ± 10%) representing the wet season in the field [39]. Pupation and emergence dates were recorded, pupae were sexed and virgin females and males were kept in different cages. We applied two feeding treatments: in the control feeding treatment, all larvae and adults were fed *ad libitum* with maize (*Zea mays*) and moist banana (*Musa acuminata*), respectively [10]. In the food stress treatment, individuals were starved at the larval stage for 48h by rearing them in a plastic petri dish with 2 cm^3^ of agar and water, while adults were fed *ad libitum*. We chose this larval food stress because it was shown to affect various life history traits in *B*. *anynana* including developmental time, pupal mass, fat percentage, resting metabolic rate and thorax ratio [40].

### Sampling collection

We collected butterfly tissues encompassing a range of experimental conditions: three different types of tissues (wings, brains, antennae), two developmental stages (pupal and adult), two food treatments (food stress and control) and the two sexes (male and female). Eight different tissues were collected from each of the feeding treatments: eight tissue samples from the control feeding and another eight from the food stress. The tissues consisted of the pupal male wing parts containing the developing androconia (Pupae M); the pupal female entire wing tissues acting as control (Pupae F); the adult male wing parts containing the androconia (Wings M And); the adult male wing parts remaining after removal of the androconia and acting as a control (Wings M Cont); the adult female entire wings acting as a third control (Wings F); the adult male (Brain M) and female (Brain F) heads after removing the antenna, eyes and proboscis; and the pooled adult male and female antennae (Antennae M, F). We produced biological replicates of each type of tissue for both the control feeding and food stress treatments by pooling the tissues of five individuals of different ages (from 1 to 14 days-old) per sample. Bulks of different tissues collected from five individuals (three biological replicates) were used for total RNA extraction. 0.5 μg of RNA from each tissue was reverse transcribed to synthesize the cDNA.

### Extraction of total RNA and cDNA synthesis

The individuals were dissected on ice directly after sacrifice and tissues were kept in RNA later (Life technologies Europe, Gent, Belgium) at -80°C according to manufacturer’s instructions for total RNA extraction. Total RNA was extracted using RNeasy Mini Kit (Qiagen Benelux) following manufacturer’s instructions. An optional step of DNaseI treatment was included during the RNA extraction process to degrade the residual DNA from the samples. The concentration and purity of the isolated RNA samples was measured using Nanodrop ND-1000 Spectrophotometer (Isogen Life Science) and RNA samples measuring the ratio OD_260_/OD_280_ in range of 1.8–2.0 were considered for RT-qPCR experiments. The integrity of isolated RNA samples was analyzed using Aligent Bioanalyzer (Aligent Biotechnologies) (S1 and S2 Figs.). First strand cDNA synthesis was carried out for each RNA sample using Superscript First-Strand Synthesis System for RT-PCR (Life technologies Europe, Gent, Belgium) following the manufacturer’s protocol in a final volume of 20 μl.

### Choice of candidate reference genes and primer design

The nucleotide sequences of the ten candidate reference genes were retrieved from the transcriptome database (http://147.99.108.61:9011/ngspipelines/#!/NGSpipelines/Bicyclus%20anynan) based on sequence homology with commonly used reference genes (Table 1). The nucleotide sequences of the genes have been submitted to NCBI Genbank database (Accessions KM923784—KM923795). The primer sequences for two genes, *eEF-1α* and *FK506* were retrieved from Pijpe *et al* [41]. For other genes, primer sequences were carefully designed using the publicly available Primer 3 software (http://bioinfo.ut.ee/primer3-0.4.0/) with the following criteria: GC content of 40–60%, Tm 60°C, length of 18–22 nucleotides and amplicon size varying between 80–120 base pairs. The secondary structures of primer sequences were checked using the software Gene Runner version 5 (www.generunner.net, version 5.0.39 Beta). Specificity of primer pairs were checked both by checking the qRT-PCR amplified products on 2% agarose gel stained in Ethidium Bromide (EtBr) and performing an *in silico* ePCR program [42].

**Table 1:** List of genes with associated functions selected for the study.

S.No.	Gene symbol	Similar to	Transcript ID	Closestortholog	E value (tBALSTN)	Gene involved in
**Candidate reference genes for normalisation**						
**1**	eEF-1α	Translation elongation factor EF-1, subunit alpha	BA_EF1A.69.80	NM_001044045.1	0.0	Regulation of transcription
**2**	FK506	FK 506 binding protein	BA_FKBP12.3.3	ABK15648.1	1e-58	Protein folding chaperones
**3**	UBQL40	ubiquitin/ribosomal protein L40 fusion protein	BA_LOC692779.3.3	NP_001037372.1	2e-87	Protein degradation
**4**	RpS8	40S ribosomal protein S8	BA_RS8.10.11	RS8_SPOFR	1e-144	Initiation of translation
**5**	GAPDH	Glyceraldehyde-3 phosphate dehydrogenase(phosphorylating)	BA_LOC692786.3.3	NP_001037386.1	0.0	Glycolysis pathway
**6**	VATPase	V-type proton ATPase subunit H	BA_VATH.3.3	VATH_MANSE	0.0	Proton gradient formation
**7**	RpS18	40S ribosomal protein S18	BA_RS18.8.8	XP_968042.1	3e-94	Initiation of translation
**8**	TBP	TATA-box-binding protein	BA_TBP.1.2	XP_969256	4e-157	Regulation of transcription
**9**	eIF2	Translation initiation factor eIF2 alpha	BA_LOC693063.1.1	NM_001044051.1	0.0	Inititation of translation
**10**	G6PD	Glucose-6-phosphate 1-dehydrogenase	BA_G6PD.2.2	G6PD_HYACE	1e-73	Pentose phosphate pathway
**11**	HSP	Hsp20/alpha crystallin family	BA_HSP21.4.18.21	NM_001043520.1	5e-16	Molecular chaperones
**12**	ACT3	Actin, cytoplasmic A3a	BA_ACT3A.1.1	ACT3A_HELAM	0.0	Cytoskeletal protein
**Gene for validating reference gene**						
**13**	FAR2	Fatty acyl reductase	BA_FACR1.6.12	JQ978771.1	0.0	Pheromone synthesis pathway
**Gene for amplifying intron sequence**						
**14**	IntFl	Elongation factor 1-alpha	BA_EF1A.38.80	EF1A_SPOFR	6e-91	Regulation of transcription

### Real-time quantitative reverse-transcription PCR (RT-qPCR)

RT-qPCR (or qRT-PCR) was performed in a 96-well thermocycler (StepOnePlus Real-Time PCR System, Software v2.1, Applied Biosystems-Life technologies) using QuantiTect SYBR Green PCR Kit (Qiagen, Benelux) and qPCR Master Mix plus one for SYBR Green I kit (Eurogentec) according to the manufacturer’s instructions. Each reaction mixture consisted of 2.5 ng of cDNA template, 0.3 μl each of forward and reverse primers, 10 μl of SBYR green mix adjusted with nuclease free water to a final volume of 20 μl. The thermocycler program included an initial denaturation step of 10 minutes at 95°C, followed by 40 cycles of 15 second at 95°C and 1 minute at 60°C. Melt curve analysis was performed after 40 cycles by heating the samples from 65°C to 95°C to confirm the specificity of primer pairs. Amplifying primer pairs for an intron sequence on cDNA checked for genomic DNA contamination. All tested samples were technically duplicated and all experiments were performed with three biological replicates.

### Data Analyses

The amplification efficiency of all primer pairs used in the study was calculated from the standard curve obtained from a five point 10-fold serial dilution series of cDNA template using the equation E (%) = (10^–1/slope^-1)*100 [43]. The expression levels of 12 candidate reference genes were calculated by Cycle threshold (Ct hereafter) values. The variations observed in the expression levels of reference genes were shown using the derived boxplot R-package BoxPlotR (http://boxplot.tyerslab.com).

The two most popular tools NormFinder [34] and geNorm [35] were used to evaluate the expression stability of all candidate reference genes across tissues and treatments (control and food stress). To meet the assumptions of the models used by NormFinder and geNorm, we used the method described in [44] and transformed the raw Ct values into 2^(-ΔCt)^ values, in which ΔCt is the difference between the Ct value of the sample tested and the minimum Ct value of the reference gene across all samples. The minimum Ct value (maximum expression level) of each gene was treated as control and was assigned a value of 1.

NormFinder uses a model based approach to assess the expression stability of different candidate reference genes by (a) measuring the overall expression level variation and (b) analyzing an intra- and intergroup variations of the reference genes. Top ranked reference genes will have minimum intra group variation and the lowest stability value. On the other hand, geNorm measures an expression stability value (M) for each reference gene by relying on the principle that the expression ratio of two ideal reference genes is identical in all samples. Thus, for every reference gene, geNorm determines the pairwise variation with all other reference genes as the standard deviation of the logarithmic transformed expression ratios, and defines the M value as the average pairwise variation of a particular reference gene with all other reference genes [35]. Moreover, Vandesompele *et al* [35] also indicated that more than one reference gene should be pooled in a “normalization factor (NF)” to best normalize qRT PCR expression data. To determine the most appropriate number of reference genes to use, the pairwise variation V_n/n+1_ can be calculated between two sequential normalization factors, *i*.*e*. between the two most stable reference genes and as many additional normalization factors as additional reference genes were tested by stepwise inclusion of the most stable remaining reference genes. A large pairwise variation means that the added reference gene has a significant effect and should preferably be included for producing a reliable normalization factor.

### Estimation of expression profile of *FAR2* gene

The relative expression profile of *FAR 2* gene was calculated in the eight different tissues (see materials and methods) using the 2^(-ΔCt)^ method. The significance of differences in gene expression levels between tissues was tested using the software R after log-transformation of the expression levels for obtaining homoscedasticity of the data across tissues, using a nested ANOVA. The independent variable, the qPCR expression levels, was compared between tissues provided that two technical replicates were produced per biological replicate and three independent biological replicates were produced per tissue type. The structure of the statistical model was thus: model <- aov (log(expression level) ~ tissue / biological replicate / technical replicate + error (tissue / biological replicate / technical replicate)).

## Results and Discussion

### Selection of candidate reference genes and amplification efficiency

Twelve reference genes commonly used in different organisms were selected as potential reference genes for *B*. *anynana*. The protein sequences of the reference genes from various insects were used as a query to perform a TBLASTN search in *B*. *anynana* transcriptome in order to obtain the sequences of the closest *B*. *anynana* orthologs. The selected candidate reference genes have various biological functions and include proteins involved in protein degradation (*UBQL40*), pentose phosphate pathway (*G6PD*), glycolysis (*GAPDH*) and ATP hydrolysis (*VATPase*). Also included are ribosomes encoding proteins (*RpS8* and *RpS18*), cytoskeletal protein (*ACT3*), transcription factor (*TBP*), heat shock protein (*HSP*) and translation initiation factor (*eIF2*) (Table 1). In addition, two reference genes previously reported in relation with ageing research in *B*. *anynana* [41], *eEF1 alpha* and *FK506* were also included in the present study (Table 1). We paid attention to selecting genes that belonged to different functional classes, which significantly reduces the chance that genes might be co-regulated. Two primer pairs were tested for each of the 12 genes and we selected for each gene the primer pair that gave the best amplification efficiency (Table 2). The amplification efficiency of primer pairs ranged between 90%-103% that suggests an efficient qRT-PCR system for *B*. *anynana*. The amplicon specificity of each primer pair was confirmed by the presence of a single melt curve peak (S3 Fig) and the presence of a single PCR amplicon of expected size on agarose gel electrophoresis (S3 Fig).

**Table 2:** Oligonucleotide sequences of genes used in the study.

S.No.	Gene symbol	Oligonucleotide sequence		Tm (°C)	Product length (bp)	E (%)	R^2^
		Forward sequence (5’- 3’)	Reverse sequence (3’- 5’)				
**1**	*eEF-1α*	GTTGAGATGCACCACGAAGC	CATAGCCACGACGCAATTCC	82,59	100	101.77	0.994
**2**	*FK 506*	AAACTAACCTGCAGCCCTGA	CAAGACGGAGAAGTTCCACA	80,05	103	90.903	0.994
**3**	*UBQL40*	CGGTAAACAATTGGAAGATGG	CGAAGTCTGAGGACAAGATGC	75,44	84	94.26	0.993
**4**	*RpS8*	GCTGAGGAAGCCATAATTAACAA	GAGGGCAGCCTCAACCTT	78,1	93	97.485	0.998
**5**	*GAPDH*	GCCCAACAGAACATCATCCC	CAACAGGTACACGGAATGCC	83,05	109	94.018	0.997
**6**	*VATPase*	CAACAGCTGTCCAAGCTGAA	CAGGGTCACAAACGAGGTCT	83,49	97	103.22	0.996
**7**	*RpS18*	TTGGATAAACGTGCTGGTGA	TGAGGAACCAGTCAGGGATT	75,41	100	95.28	0.991
**8**	*TBP*	CTGCTCGGAAATATGCAAGG	GACATCACAACTGCCAACCA	83,3	95	98.254	0.997
**9**	*eIF2*	TAAAGTGGTGACGGCGACAG	CTCGTCAGCCGAGTCTCC	80,05	97	101.72	0.993
**10**	*G6PD*	CTTCGCGCAAACAGAATCTT	CGCGCGTATATCCTTTAATAGC	74,41	106	96.413	0.991
**11**	*HSP*	TCTGGATCGGGATGTTCCTA	ACAAACCCAGAGGCAATCAA	82,41	106	102.48	0.993
**12**	*ACT3*	AAGATCATCGCTCCTCCAGA	CGGACTCGTCGTACTCCTGT	81,95	115	93.746	0.998
**13**	*FAR2*	TGATGCGCAAGTCAAAGAAC	AGGTACATGCCCATGGTTGT	77.67	103	101.08	0.999
**14**	*IntFl*	CTGCAAGTTTGCCGAGATTA	GCCCGACTTGATCGACTTC	79.24	85*/580**	97.33	0.991

Tm: Melting temperature; bp: base pairs; E: efficiency of primer; R^2^: Regression coefficient.

* indicates the amplicon size amplified on cDNA

** indicates the amplicon size amplified on genomic DNA.

### Expression profile of the 12 candidate reference genes

**Fig 1:**
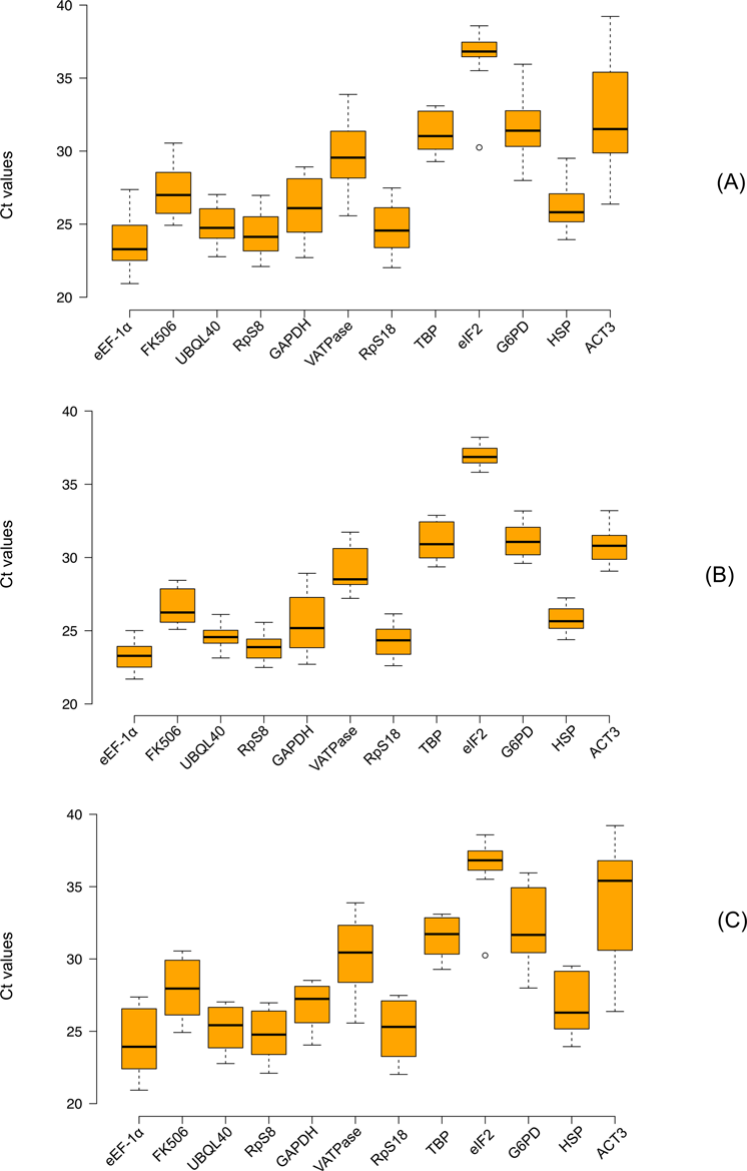
Expression range of Ct values of 12 candidate reference genes. (A) total tissues, n = 8 sample points (B) control tissues, n = 8 sample points and (C) food stressed tissues, n = 16 sample points. Black line across each box represents the median. Box limits indicate the 25^th^ and 75^th^ percentiles as determined by R software; whiskers extend 1.5 times the interquartile range from the 25^th^ and 75^th^ percentiles, outliers are represented by dots. Ct values and genes are shown on Y- and X-axis, respectively.

RT-qPCR was carried out to measure the transcript abundance of the candidate reference genes in a set of different types of tissues, developmental stages, food treatments and sexes (see Material and Methods). The potential reference genes exhibited a wide variability in expression profile under both experimental conditions (food stress and control feeding *ad libitum*). Overall, the observed Ct values for the 12 genes ranged from a minimum Ct value of 20.92 (*eEF-1α*) to maximum Ct value of 39.21 (*ACT3*) (Fig. 1A). The gene *eIF2* showed least average transcript abundance in control and food stress treatments (Ct = 36.95± 0.26 and 36.21± 0.42 cycles, mean± standard deviation, respectively) and it showed minimum cycle of variation of 2.4 and 3.8 cycles in control and food stress treatments, respectively. The two most abundant genes were *eEF-1α* (Ct = 23.27± 0.24 and 24.26± 0.26 cycles, respectively) and *RpS8* (Ct = 23.87± 0.42 and 24.77± 0.29 cycles, respectively) and showed moderate variation in Ct range of 3.3 and 3.1 cycles respectively (Fig. 1A).

When the gene expression profiles across eight different tissues were analyzed separately for control (Fig. 1B) and food stress treatments (Fig. 1C) seven genes, namely *eEF-1α*, *UBQL40*, *RpS8*, *RpS18*, *GAPDH*, *Act3* and *G6PD* showed a significant Ct variation (p value < 0.05; Ansari-Bradley Non-parametric homoscedasticity test) in food stress treatment when compared with control treatment (S4 Fig). For the remaining five reference genes (*FK506*, *HSP*, *VATPase* and *HSP*), we did not observe a statistically significant variation between the food stress and control treatments. The variation in Ct range fell between 2.4 to 6.2 cycles for all candidate reference genes for the control treatment (Fig. 1B), and increased between 3.8 to 12.8 cycles in the food stress treatment (Fig. 1C and S1 Data). Thus, no candidate reference gene had a very stable expression level across the tested tissues and treatments.

### Expression stability of candidate reference genes

We used the two most commonly used statistical programs NormFinder and geNorm to monitor the expression stability of twelve candidate reference genes. Zhu *et al* [45] showed that these two programs are sufficient to select the appropriate reference genes.

NormFinder calculated the stability values of the 12 genes and ranked the genes accordingly (Table 3). When NormFinder analysis was applied to pooled control and food stress treatments, the most stable reference gene was *FK506* (M value = 0.21) followed by *RpS8* and *RpS18* (Table 3). In the control feeding treatment, the most stable reference gene was *RpS8* (M value = 0.22) followed by *FK506* and *UBQL40* (Table 3). In the food stressed treatment, *FK506* with an M value of 0.13 was the best choice for a reference gene, followed by *RpS18* and *RpS8* (Table 3). In both control and food stressed conditions, *ACT3* was the least stable reference gene.

**Table 3:** Expression stability of candidate reference genes ranked by NormFinder in different tissue sets.

	All tissues		Control tissues		Food stressed tissues	
Rank	Gene name	Stability value (M)	Gene name	Stability value (M)	Gene name	Stability value (M)
**1**	*FK506*	0,21	*RpS8*	0,225	*FK506*	0,130
**2**	*RpS8*	0,25	*FK506*	0,239	*RpS18*	0,314
**3**	*RpS18*	0,33	*UBQL40*	0,270	*RpS8*	0,321
**4**	*HSP*	0,34	*HSP*	0,330	*HSP*	0,342
**5**	*UBQL40*	0,35	*RpS18*	0,364	*UBQL40*	0,409
**6**	*eEF-1α*	0,45	*G6PD*	0,369	*eEF-1α*	0,498
**7**	*VATPase*	0,47	*eEF-1α*	0,395	*VATPase*	0,514
**8**	*G6PD*	0,59	*TBP*	0,410	*GAPDH*	0,641
**9**	*TBP*	0,61	*VATPase*	0,474	*TBP*	0,733
**10**	*GAPDH*	0,90	*eIF2*	0,544	*G6PD*	0,787
**11**	*eIF2*	1,00	*GAPDH*	1,136	*eIF2*	1,010
**12**	*ACT3*	1,96	*ACT3*	1,256	*ACT3*	2,468

**Fig 2:**
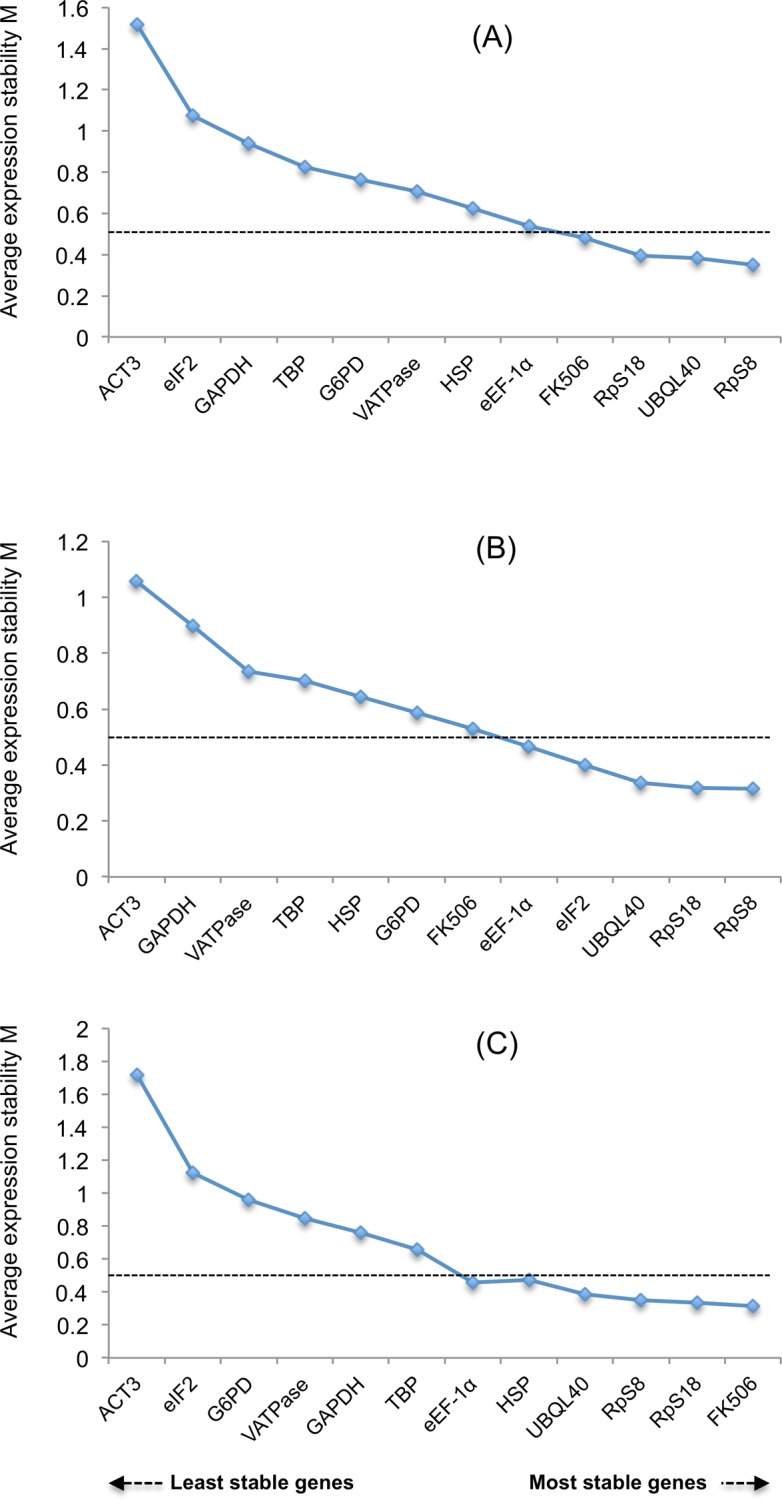
Measures of expression stability (M value). geNorm analysis and ranking of 12 candidate reference genes in (A) all tissues (B) control tissues and (C) food stressed tissues. Suitable reference genes are assigned M values below 0.5 (dotted line).

Second, we used geNorm, for which Vandesompele *et al* [35] strongly recommends a threshold expression stability M value of 0.5. When M values from both control and food stressed treatments were collectively evaluated, four out of twelve reference genes showed M value lower than 0.5, and *RpS8* had the lowest M value of 0.35 and therefore was the best reference gene (Fig. 2A). In the control treatment, 6 out of 12 reference genes had a M value lower than 0.5 (Fig. 2B) while in the food stressed treatment, five out of twelve tested reference genes had a M value lower than 0.5 (Fig. 2C), among which, *RpS8* was each time the most stable reference gene with an acceptable M value of 0.31. Our earlier conclusion that *ACT3* was the least stable reference gene was also supported by geNorm analysis.

Thus, both NormFinder and geNorm propose the ribosomal protein *RpS8* as the best reference gene under the control treatment (Table 4). Ribosomal proteins are abundant in different tissues at all different stages and therefore various ribosomal proteins have been successfully selected and validated as best reference genes in insects [23–25,46–48]. In the food stressed treatment, both software packages ranked *FK506* as the best choice of reference gene. Our findings are in partial agreement with of a previous study by Pijpe *et al* [41] that monitored five reference genes in *B*. *anynana*: while *FK506* was previously found as one of the most suitable reference gene for normalizing expression analysis data of tissues under food stress in *B*. *anynana* [41], *eEF-1α* that appeared to be the best choice as a reference gene in Pijpe *et al* [41], displayed a low ranking (fifth position) in our study (Table 4). Two reasons can explain the discrepancies observed between our and Pijpe *et al*’s results: first, we used a larger number of candidate genes that allowed us to find a reference gene, *RpS8*, that outcompeted *FK506* in some conditions; second, Pijpe et al’s study ranked the candidate genes using the geNorm software only, while it is strongly recommended to use both geNorm and NormFinder to identify the best reference genes [49].

**Table 4:** Consensus ranking of candidate reference genes.

	All tissues					Control tissues					Food stressed tissues				
Gene	geNorm	Rank	NormFinder	Rank	Consensus*	geNorm	Rank	NormFinder	Rank	Consensus*	geNorm	Rank	NormFinder	Rank	Consensus*
*RpS8*	0,350	1	0,250	2	1	0,314	1	0,225	1	1	0,349	3	0,321	3	3
*FK506*	0,480	4	0,210	1	2	0,529	6	0,239	2	4	0,314	1	0,130	1	1
*UBQL40*	0,384	2	0,350	5	3	0,336	3	0,270	3	3	0,388	4	0,409	5	4
*HSP*	0,625	6	0,340	4	5	0,645	8	0,330	4	6	0,474	5	0,342	4	4
*RpS18*	0,396	3	0,330	3	3	0,317	2	0,364	5	3	0,334	2	0,314	2	2
*G6PD*	0,765	8	0,590	8	8	0,587	7	0,369	6	6	0,958	10	0,787	10	10
*eEF-1α*	0,536	5	0,450	6	5	0,466	5	0,395	7	6	0,456	6	0,498	6	6
*TBP*	0,825	9	0,610	9	9	0,702	9	0,410	8	8	0,658	7	0,733	9	8
*VATPase*	0,705	7	0,470	7	7	0,735	10	0,474	9	9	0,849	9	0,514	7	8
*eIF2*	1,073	11	1,000	11	11	0,399	4	0,544	10	7	1,121	11	1,010	11	11
*GAPDH*	0,940	10	0,900	10	10	0,897	11	1,136	11	11	0,760	8	0,641	8	8
*ACT3*	1,517	12	1,960	12	12	1,057	12	1,256	12	12	1,717	12	2,468	12	12
**Best gene**	***RpS8***		***RpS8***		***RpS8***		***RpS8***	***RpS8***		***RpS8***	***FK506***		***FK506***		***FK506***
**Best gene Combination**	***Rps8/FK506***					***RpS8/RpS18***					***FK506/RpS18***				

*The ranking has been calculated by averaging the ranks obtained by geNorm and NormFinder.

Although actin proteins are used as reference genes in honeybee *Apis mellifera* [50] and in desert locus *Schistocerca gregaria* [51], our analysis ranked actin (*ACT3*) as the least stable reference gene. Our results confirm that actin appears to be one of the most unsuitable choices for RT-qPCR data normalization in various taxa [52]. Similarly, *GAPDH* that showed maximum stability as a reference gene in the moths *Bombyx mori* and *Spodoptera exigua* [25], was however not selected as a stable reference gene in our study. It appears that traditional reference genes like Actin and *GAPDH* may not be the best choice as normalizing genes for all organisms. Consequently, our results show that new reference genes can outperform the traditional reference genes and signifies the importance of identifying and validating suitable reference gene in *B*. *anynana*.

**Fig 3:**
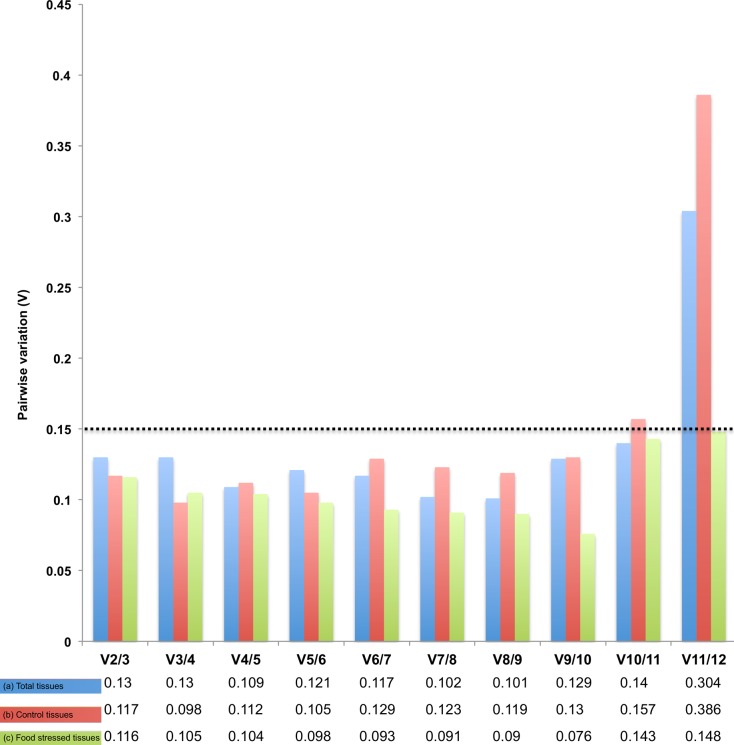
Determination of the suitable number of reference genes for normalization. Pairwise variation (V) was calculated for (a) total tissues (b) control tissues and (c) food stressed tissues for the 12 candidate reference genes using geNorm. Dotted lines represent the recommended threshold in [35].

Although several of the 12 candidate genes showed sufficiently stable expression across different tissues to be used as reference genes in *B*. *anynana* expression analyses, we further tested whether the use of more than one reference gene could improve the normalization of the qRT-PCR expression data. We used geNorm that calculates the pairwise variation (V_n_/V_n+1_) between groups of reference genes that are ranked according to their expression stability across samples (see materials and methods). In both our experimental treatments (food stress and control feeding *ad libitum*), geNorm provided a V2/3 below the cut off value of 0.15 indicating that the use of a maximum of two best reference genes should accurately normalize the expression results (Fig. 3). For control tissues, the combination of the two reference genes *RpS8* and *RpS18* (V2/3 = 0.116) could adequately normalize the expression data. Similarly, *RpS18* with *FK506* (V2/3 = 0.117) was sufficient for a correct quantification for tissues under food stress. Similarly, there is no increase in the optimal number of reference genes required for accurate normalization when tissues under both conditions were collectively analyzed and reference genes *Rps8* with *UBQL40* (V2/3 = 0.13) appeared the best for data normalization (Fig. 3). Thus, *Rps8* and *Rps18* were recurrently selected as part of the best pair of reference genes across our range of experimental tissues and treatments. Yet, because different pairs of reference genes were found to form the best combination in different treatments, we strongly recommend evaluating the identity of the best reference genes according to the specific experimental requirements and conditions.

### Validation of the choice of reference genes using a real case study in sex pheromone biosynthesis

**Fig 4:**
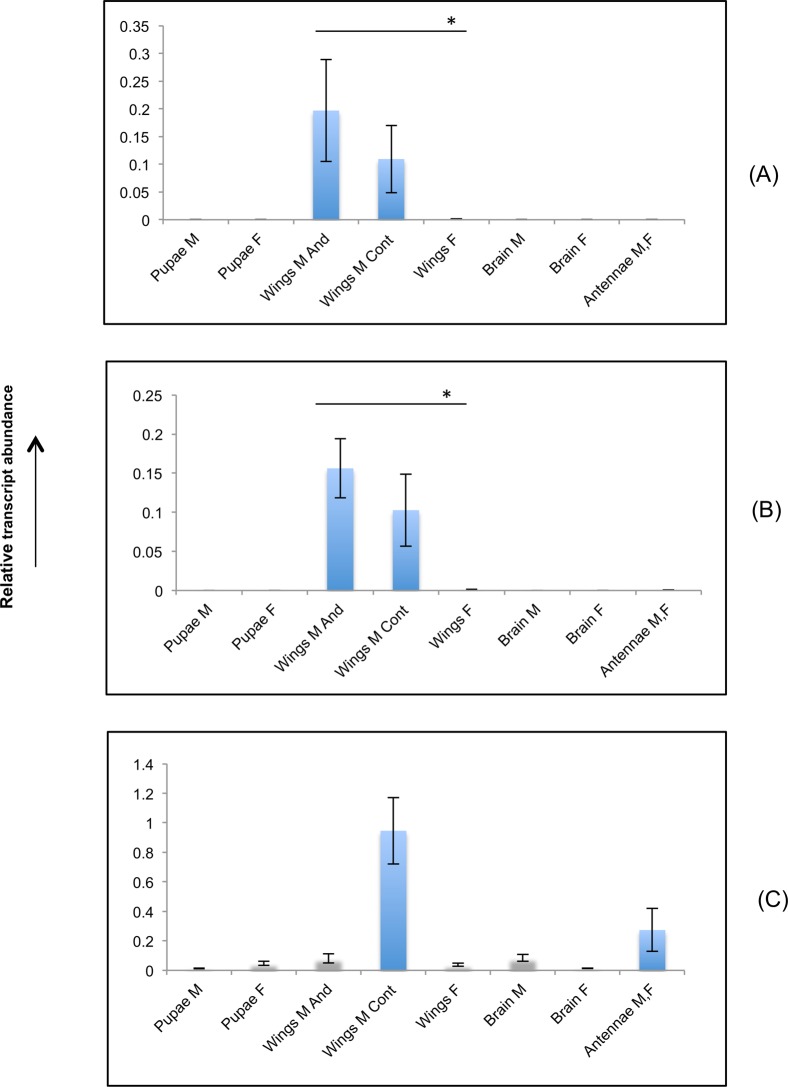
Relative expression profile of *FAR2* gene. Effect of normalization with (A) *RpS8*, (B) *Rps8* and *RpS18*, and(C) *ACT3* on the expression of *FAR 2* gene. Data are means of three biological replicates ± SE. Significant difference in the transcript abundance of male wing tissue containing the androconia (“Wings M And”) as compared with female wing tissues (“Wings F”) was observed in (A) and (B) (* indicates p-value < 0.01). Pupae M = pupae male; Pupae F = pupae female; Wings M And = adult wings male androconia; Wings M Cont = Adult wings male control; Wings F = adult wings female; Brain M = Brain male; Brain F = Brain female; Antennae M,F = antennae male and female.

To evaluate the effect of choosing a least or most stably expressed reference gene on the normalization of expression profile of genes, we measured the transcript abundance of the Fatty Acyl Reductase 2 gene (*Ban-wFAR2* in [36]), a gene shown to be involved in the biosynthesis of the male sex pheromone component Z9-tetradecen-1-ol (MSP1 hereafter) in *B*. *anynana* [36]. We normalized the expression results for *FAR2* using the best (*Rps8*) and least stable (*ACT3*) reference genes as ranked by NormFinder (Table 4). When *Rps8* was used, the *FAR2* transcript was significantly more expressed in male wing androconial tissues that produce sex pheromone [14,36], compared to control adult male and female wing tissues (Fig. 4A) that do not display the androconial tissues and display a significant lower abundance of MSP1 [14]. The expression of *FAR2* was absent in all other tissues (Fig. 4A). Similar results were obtained when the data was normalized using the combination of the two best genes suggested by geNorm (*RpS8* and *RpS18*; Fig. 4B). In contrast, when the least stable *ACT3* candidate reference was used to normalize the expression pattern, we observed an overexpression of *FAR2* in the male control tissues while *FAR2* appeared less expressed in other tissues (Fig. 4C). Since androconial wing tissues are the primary sites of sex pheromone biosynthesis in *B*. *anynana* [14], the presence of very low expression of *FAR2* in this tissue as compared to control tissues is unexpected. Similarly, the expression of *FAR2* in the antennal tissues (Fig. 4C) is highly unlikely as there is no MSP1 in the antenna [53]. Consequently, we confirm for *B*. *anynana* that the choice of a reference gene to normalize the relative expression profile of genes of interest can alter the final outcome of the expression analysis and should be the object of a careful selection.

## Conclusion

To the best of our knowledge, our work is the first in-depth study to identify and validate reference genes for qRT-PCR data normalization in a butterfly. In the present study, twelve candidate reference genes were identified and their expression profiles were measured across different tissues and conditions to identify the best reference genes in *B*. *anynana* for qRT-PCR data normalization. Ribosomal protein *RpS8* was identified as the best choice of reference gene for normalizing gene expression data of genes involved in olfactory communication. Additionally, we found that actin *ACT3* being the least stable should not be used as a choice for an internal control. We expect an increased interest in the lepidopteron community to assess the expression profile of gene families in *B*. *anynana* and compare it to other butterflies.

## Supporting Information

S1 Fig RNA quality test for samples of control treatment. (TIF)

S2 Fig RNA quality test for samples of food stressed treatment. (TIF)

S3 Fig Amplicon specificity. (TIF)

S4 Fig Comparison of variance of two treatments. (TIF)

S1 Data Ct values of all samples. (XLSX)

## References

[1] Boppré M (1984) The Biology of Butterflies Vanewright R., Ackey P., editors Academic Press.

[2] Scoble M (1992) The Lepidoptera, Form, Function and Diversity. Oxford: Oxford University Press.

[3] Wahlberg N, Wheat CW, Peña C (2013) Timing and patterns in the taxonomic diversification of Lepidoptera (butterflies and moths). PLoS One 8: e80875 10.1371/journal.pone.0080875 24282557 PMC3839996

[4] Consortium THG (2012) Butterfly genome reveals promiscuous exchange of mimicry adaptations among species. Nature 487: 94–98. 10.1038/nature11041 22722851 PMC3398145

[5] Zhan S, Merlin C, Boore JL, Reppert SM (2011) The monarch butterfly genome yields insights into long-distance migration. Cell 147: 1171–1185. 10.1016/j.cell.2011.09.052 22118469 PMC3225893

[6] Ahola V, Lehtonen R, Somervuo P, Salmela L, Koskinen P, Rastas P, et al (2014) The Glanville fritillary genome retains an ancient karyotype and reveals selective chromosomal fusions in Lepidoptera. Nat Commun 5: 4737 10.1038/ncomms5737 25189940 PMC4164777

[7] Beldade P, McMillan WO, Papanicolaou A (2008) Butterfly genomics eclosing. Heredity (Edinb) 100: 150–157. 17290215 10.1038/sj.hdy.6800934

[8] Zhu H, Casselman A, Reppert SM (2008) Chasing migration genes: a brain expressed sequence tag resource for summer and migratory monarch butterflies (*Danaus plexippus*). PLoS One 3: e1345 10.1371/journal.pone.0001345 18183285 PMC2156104

[9] Brunetti CR, Selegue JE, Monteiro A, French V, Brakefield PM, Carroll SB. (2001) The generation and diversification of butterfly eyespot color patterns. Curr Biol 11: 1578–1585. 11676917 10.1016/s0960-9822(01)00502-4

[10] Brakefield PM, Beldade P, Zwaan BJ (2009) The African Butterfly *Bicyclus anynana*: A Model for Evolutionary Genetics and Evolutionary Developmental Biology. Cold Spring Harb Protoc. 10.1101/pdb.emo122 20147150

[11] Beldade P, Brakefield PM (2002) The genetics and evo-devo of butterfly wing patterns. Nat Rev Genet 3: 442–452. 12042771 10.1038/nrg818

[12] San Martin Y Gomez G, Bacquet P, Nieberding C (2011) Mate choice and sexual selection in a model butterfly species, *Bicyclus anynana*: state of the art. Netherlands Entomological Society Meeting. Proceedings. pp. 9–22.

[13] Van Bergen E, Brakefield PM, Heuskin S, Zwaan BJ, Nieberding CM (2013) The scent of inbreeding: a male sex pheromone betrays inbred males. Proc R Soc B Biol Sci 280. 10.1098/rspb.2013.0102 PMC3619463 23466986

[14] Nieberding CM, de Vos H, Schneider M V, Lassance J-M, Estramil N, Andersson J, et al (2008) The male sex pheromone of the butterfly *Bicyclus anynana*: towards an evolutionary analysis. PLoS One 3: e2751 10.1371/journal.pone.0002751 18648495 PMC2447158

[15] Nieberding CM, Fischer K, Saastamoinen M, Allen CE, Wallin EA, Hedenström E, et al (2012) Cracking the olfactory code of a butterfly: the scent of ageing. Ecol Lett 15: 415–424. 10.1111/j.1461-0248.2012.01748.x 22390373

[16] Condamin M (1973) Monographie du genre Bicyclus (Lepidoptera, Satyridae). Dakar: Institut fondamental d’Afrique noire.

[17] Robertson KA, Monteiro A (2005) Female *Bicyclus anynana* butterflies choose males on the basis of their dorsal UV-reflective eyespot pupils. Proceedings Biol Sci R Soc 272: 1541–1546. 16048768 10.1098/rspb.2005.3142 PMC1559841

[18] Bacquet PMB, Brattström O, Wang H-L, Allen CE, Löfstedt C, Brakefield PM, et al (2015) Selection on male sex pheromone composition contributes to butterfly reproductive isolation. Proc R Soc B Biol Sci: In press. 10.1098/rspb.2014.2734 PMC4375863 25740889

[19] Gutierrez L, Mauriat M, Guénin S, Pelloux J, Lefebvre J-F, Louvet R, et al (2008) The lack of a systematic validation of reference genes: a serious pitfall undervalued in reverse transcription-polymerase chain reaction (RT-PCR) analysis in plants. Plant Biotechnol J 6: 609–618. 10.1111/j.1467-7652.2008.00346.x 18433420

[20] Czechowski T, Stitt M, Altmann T, Udvardi MK, Scheible W-R (2005) Genome-wide identification and testing of superior reference genes for transcript normalization in *Arabidopsis* . Plant Physiol 139: 5–17. 16166256 10.1104/pp.105.063743 PMC1203353

[21] Kuijk EW, du Puy L, van Tol HT, Haagsman HP, Colenbrander B, Bernard AJ, et al (2007) Validation of reference genes for quantitative RT-PCR studies in porcine oocytes and preimplantation embryos. BMC Dev Biol 7: 58 17540017 10.1186/1471-213X-7-58 PMC1896162

[22] Ponton F, Chapuis M-P, Pernice M, Sword GA, Simpson SJ (2011) Evaluation of potential reference genes for reverse transcription-qPCR studies of physiological responses in *Drosophila melanogaster* . J Insect Physiol 57: 840–850. 10.1016/j.jinsphys.2011.03.014 21435341

[23] Zhu X, Yuan M, Shakeel M, Zhang Y, Wang S, Wang X, et al (2014) Selection and evaluation of reference genes for expression analysis using qRT-PCR in the beet armyworm *Spodoptera exigua* (Hübner) (Lepidoptera: Noctuidae). PLoS One 9: e84730 10.1371/journal.pone.0084730 24454743 PMC3893131

[24] Wang G-H, Xia Q-Y, Cheng D-J, Duan J, Zhao P, Chen J, et al (2008) Reference genes identified in the silkworm *Bombyx mori* during metamorphism based on oligonucleotide microarray and confirmed by qRT-PCR. Insect Sci 15: 405–413.

[25] Teng X, Zhang Z, He G, Yang L, Li F (2012) Validation of reference genes for quantitative expression analysis by real-time rt-PCR in four lepidopteran insects. J Insect Sci 12: 60 10.1673/031.012.6001 22938136 PMC3481461

[26] Fu W, Xie W, Zhang Z, Wang S, Wu Q, Liy Y, et al (2013) Exploring valid reference genes for quantitative real-time PCR analysis in *Plutella xylostella* (Lepidoptera: Plutellidae). Int J Bol Sci 9: 792–802. 10.7150/ijbs.5862 PMC3753443 23983612

[27] Zhang S, An S, Li Z, Wu F, Yang Q, Liu Y, et al (2015) Identification and validation of reference genes for normalization of gene expression analysis using qRT-PCR in *Helicoverpa armigera* (Lepidoptera: Noctuidae). Gene 555: 393–402. 10.1016/j.gene.2014.11.038 25447918

[28] Gu S-H, Wu K-M, Guo Y-Y, Pickett JA, Field LM Zhou J-J, et al (2013) Identification of genes expressed in the sex pheromone gland of the black cutworm *Agrotis ipsilon* with putative roles in sex pheromone biosynthesis and transport. BMC Genomics 14: 636 10.1186/1471-2164-14-636 24053512 PMC3849270

[29] Lu Y, Yuan M, Gao X, Kang T, Zhan S, Wan H, et al (2013) Identification and validation of reference genes for gene expression analysis using quantitative PCR in *Spodoptera litura* (Lepidoptera: Noctuidae). PLoS One 8: e68059 10.1371/journal.pone.0068059 23874494 PMC3706614

[30] Ometto L, Shoemaker D, Ross KG, Keller L (2011) Evolution of gene expression in fire ants: the effects of developmental stage, caste, and species. Mol Biol Evol 28: 1381–1392. 10.1093/molbev/msq322 21172833

[31] Haskins WE, Sponsel V, Cassill A, Renthal R (2009) The major antennal chemosensory protein of red. Insect Mol Biol 18: 395–404. 10.1111/j.1365-2583.2009.00883.x 19523071 PMC2771726

[32] Dong M, Zhang X, Chi X, Mou S, Xu J, Xu D, et al (2012) The validity of a reference gene is highly dependent on the experimental conditions in green alga *Ulva linza* . Curr Genet 58: 13–20. 10.1007/s00294-011-0361-3 22205301

[33] Teste M-A, Duquenne M, François JM, Parrou J-L (2009) Validation of reference genes for quantitative expression analysis by real-time RT-PCR in *Saccharomyces cerevisiae* . BMC Mol Biol 10: 99 10.1186/1471-2199-10-99 19874630 PMC2776018

[34] Andersen CL, Jensen JL, Ørntoft TF (2004) Normalization of Real-Time Quantitative Reverse Transcription-PCR Data: A Model-Based Variance Estimation Approach to Identify Genes Suited for Normalization, Applied to Bladder and Colon Cancer Data Sets Normalization of Real-Time Quantitative Reverse. Cancer Res 64: 5245–5250. 15289330 10.1158/0008-5472.CAN-04-0496

[35] Vandesompele J, De Preter K, Pattyn F, Poppe B, Van Roy N, De Paepe A, et al (2002) Accurate normalization of real-time quantitative RT-PCR data by geometric averaging of multiple internal control genes. Genome Biol 3: 1–11. 10.1186/gb-2002-3-7-research0034 PMC126239 12184808

[36] Liénard MA, Wang H-L, Lassance J-M, Löfstedt C (2014) Sex pheromone biosynthetic pathways are conserved between moths and the butterfly *Bicyclus anynana* . Nat Commun 5: 3957 10.1038/ncomms4957 24862548 PMC4050330

[37] Brakefield PM, Reitsma N (1991) Phenotypic plasticity, seasonal climate and the population biology of *Bicyclus* butterflies (Satyridae) in Malawi. Ecol Entomol 16: 291–303.

[38] Van’t Hof AE, Marec F, Saccheri IJ, Brakefield PM, Zwaan BJ (2008) Cytogenetic characterization and AFLP-based genetic linkage mapping for the butterfly *Bicyclus anynana*, covering all 28 karyotyped chromosomes. PLoS One 3: e3882 10.1371/journal.pone.0003882 19060955 PMC2588656

[39] Brakefield PM, Kesbeke F, Koch PB (1998) The regulation of phenotypic plasticity of eyespots in the butterfly *Bicyclus anynana* . Am Nat 152: 853–860. 10.1086/286213 18811432

[40] Saastamoinen M, Brommer JE, Brakefield PM, Zwaan BJ (2013) Quantitative genetic analysis of responses to larval food limitation in a polyphenic butterfly indicates environment- and trait-specific effects. Ecol Evol 3: 3576–3589. 10.1002/ece3.718 24223292 PMC3797501

[41] Pijpe J, Pul N, van Duijn S, Brakefield PM, Zwaan BJ (2011) Changed gene expression for candidate ageing genes in long-lived *Bicyclus anynana* butterflies. Exp Gerontol 46: 426–434. 10.1016/j.exger.2010.11.033 21118714

[42] Schuler GD (1997) Sequence Mapping by Electronic PCR Sequence Mapping by Electronic PCR. Genome Res: 541–550. 9149949 10.1101/gr.7.5.541 PMC310656

[43] Radonić A, Thulke S, Mackay IM, Landt O, Siegert W, Nitsche A. (2004) Guideline to reference gene selection for quantitative real-time PCR. Biochem Biophys Res Commun 313: 856–862. 14706621 10.1016/j.bbrc.2003.11.177

[44] Pfaffl MW (2001) A new mathematical model for relative quantification in real-time RT-PCR. Nucleic Acids Res 29: 45e—45. 10.1093/nar/29.9.e45 PMC55695 11328886

[45] Zhu X, Li X, Chen W, Chen J, Lu W, Chen L, et al (2012) Evaluation of new reference genes in papaya for accurate transcript normalization under different experimental conditions. PLoS One 7: e44405 10.1371/journal.pone.0044405 22952972 PMC3432124

[46] Cheng D, Zhang Z, He X, Liang G (2013) Validation of reference genes in *Solenopsis invicta* in different developmental stages, castes and tissues. PLoS One 8: e57718 10.1371/journal.pone.0057718 23469057 PMC3585193

[47] Groot A, Schöfl G, Inglis O, Donnerhacke S, Classen A, Schmalz A, et al (2014) Within-population variability in a moth sex pheromone blend: genetic basis and behavioural consequences. Proc R Soc London B Biol Sci 281: 20133054. 10.1098/rspb.2013.3054 PMC3924083 24500170

[48] Lord J, Hartzer K, Toutges M, Oppert B (2010) Evaluation of quantitative PCR reference genes for gene expression studies in *Tribolium castaneum* after fungal challenge. J Microbiol Methods 80: 219–221. 10.1016/j.mimet.2009.12.007 20026205

[49] Kong Q, Yuan J, Niu P, Xie J, Jiang W, Huang Y, et al (2014) Screening suitable reference genes for normalization in reverse transcription quantitative real-time PCR analysis in melon. PLoS One 9: e87197 10.1371/journal.pone.0087197 24475250 PMC3903635

[50] Lourenco P, Aline M, Cristino A dos S, Simoes ZLP (2008) Validation of reference genes for gene expression studies in the honey bee, *Apis mellifera*, by quantitative real-time RT-PCR. Apidologie 39: 372–385.

[51] Van Hiel MB, Van Wielendaele P, Temmerman L, Van Soest S, Vuerinckx K, Huybrechts R, et al (2009) Identification and validation of housekeeping genes in brains of the desert locust *Schistocerca gregaria* under different developmental conditions. BMC Mol Biol 10: 56 10.1186/1471-2199-10-56 19508726 PMC2700112

[52] Huggett J, Dheda K, Bustin S, Zumla A (2005) Real-time RT-PCR normalisation; strategies and considerations. Genes Immun 6: 279–284. 15815687 10.1038/sj.gene.6364190

[53] Heuskin S, Vanderplanck M, Bacquet P, Holveck M-J, Kaltenpoth M, Engl T, et al (2014) The composition of cuticular compounds indicates body parts, sex and age in the model butterfly *Bicyclus anynana* (Lepidoptera). Front Ecol Evol 2: 1–16.

