# Supplementary figures and images for: Selection and Validation of Reference Genes for qRT-PCR Expression Analysis of Candidate Genes Involved in Olfactory Communication in the Butterfly Bicyclus anynana
Source: PLoS One. 2015 Mar 20;10(3):e0120401. doi: 10.1371/journal.pone.0120401 (PMC4368739; doi:10.1371/journal.pone.0120401)

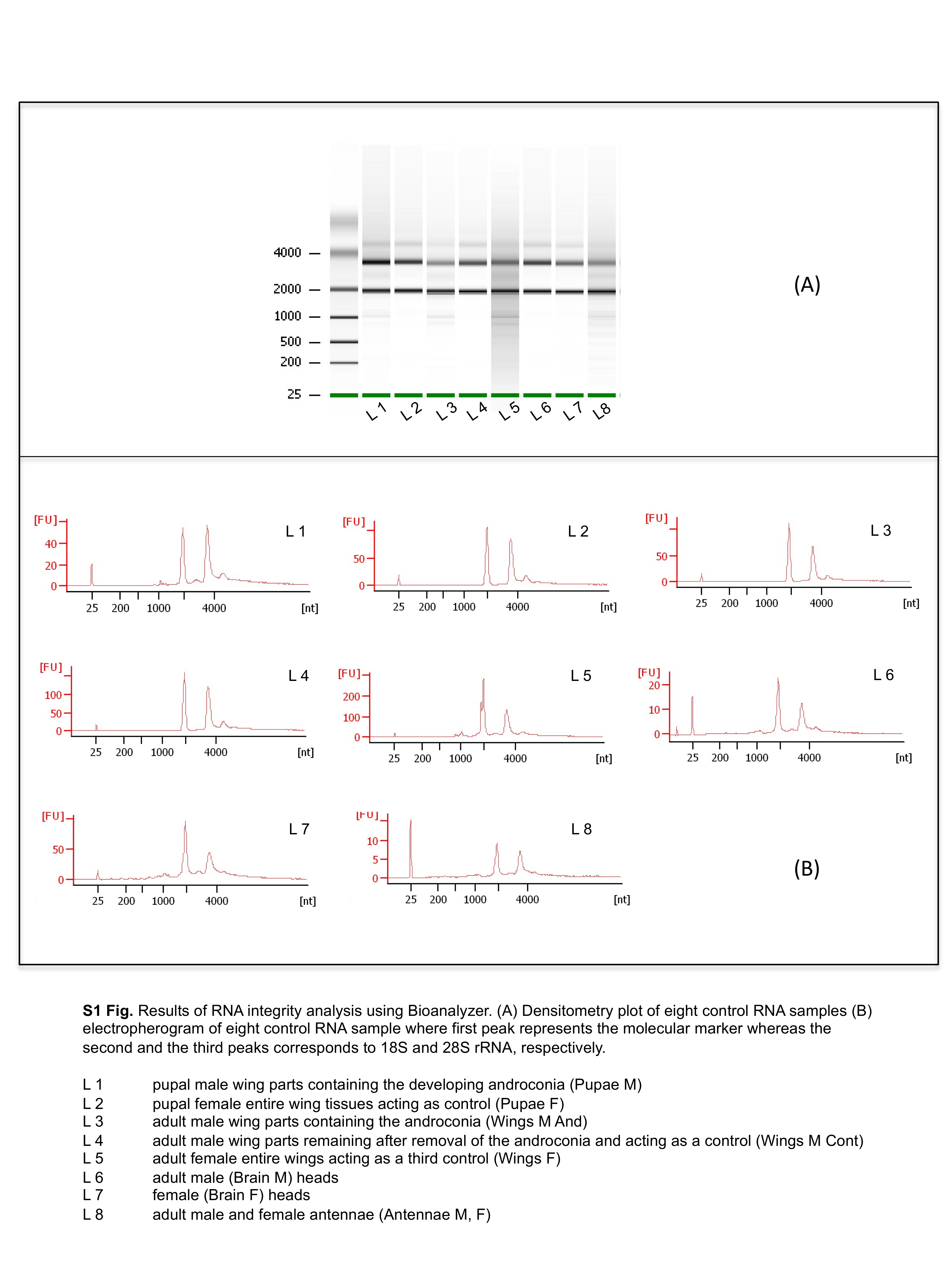

Supplement: S1 Fig — (TIF) [file pone.0120401.s001.tif]

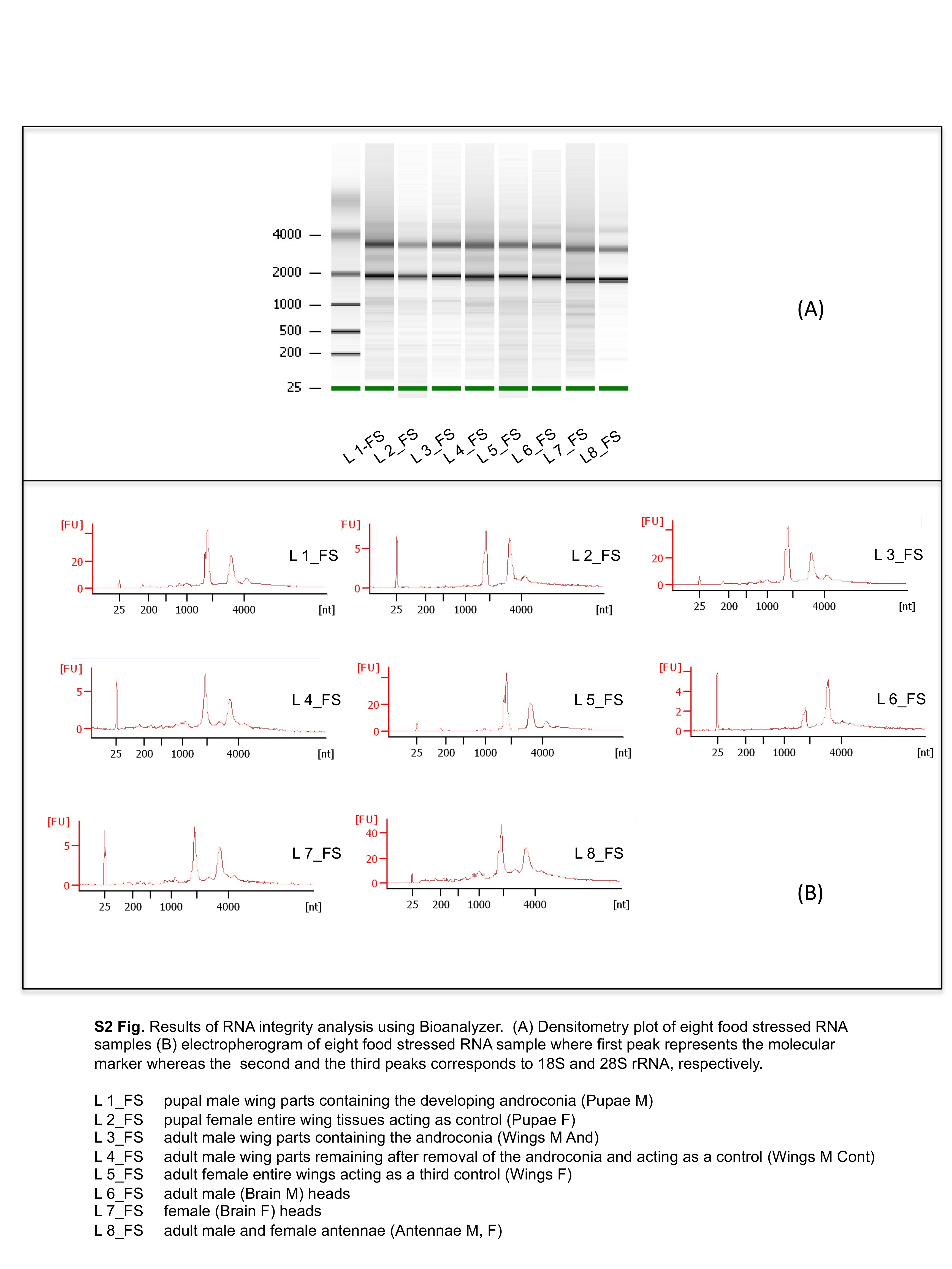

Supplement: S2 Fig — (TIF) [file pone.0120401.s002.tif]

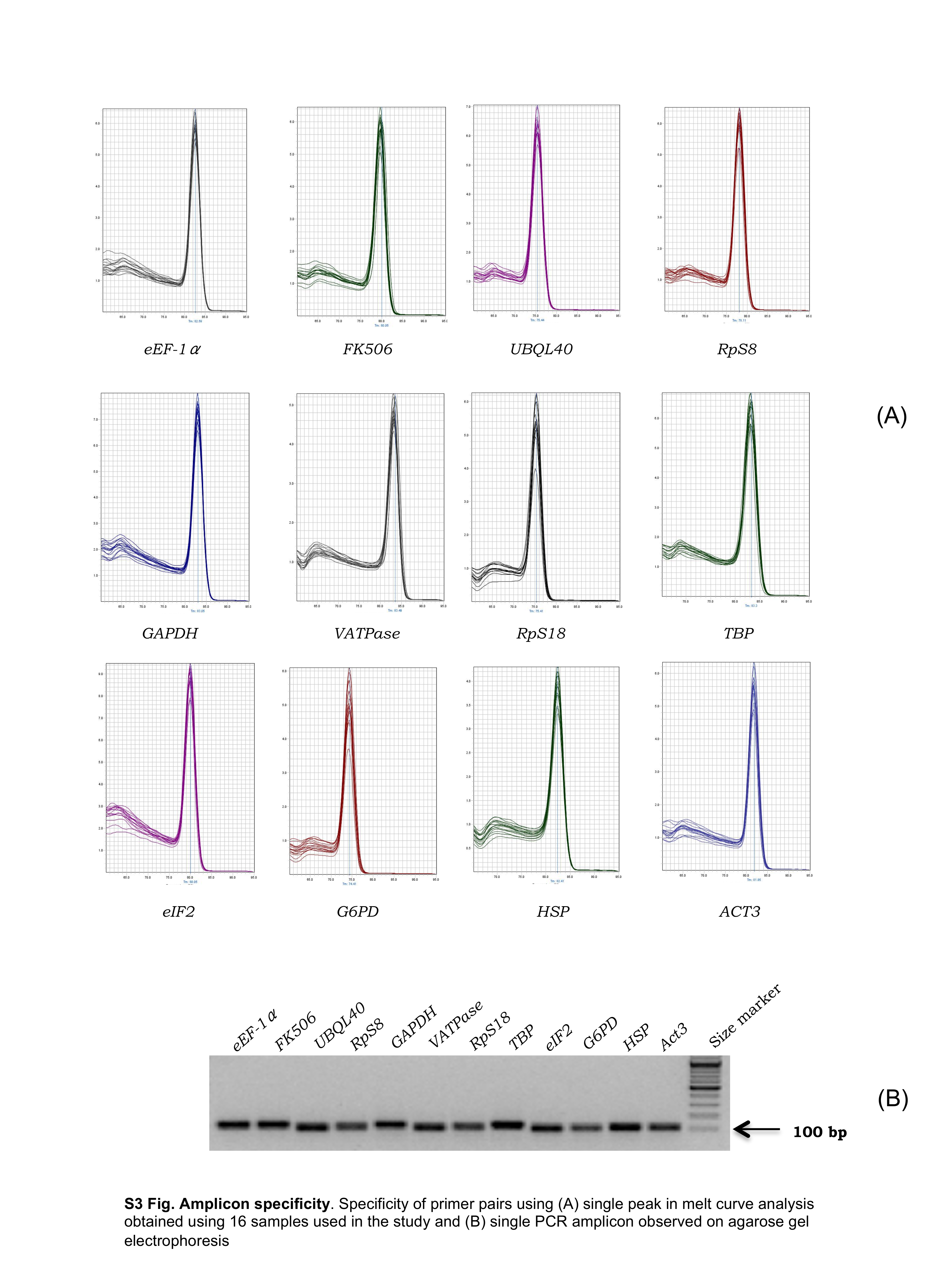

Supplement: S3 Fig — (TIF) [file pone.0120401.s003.tif]

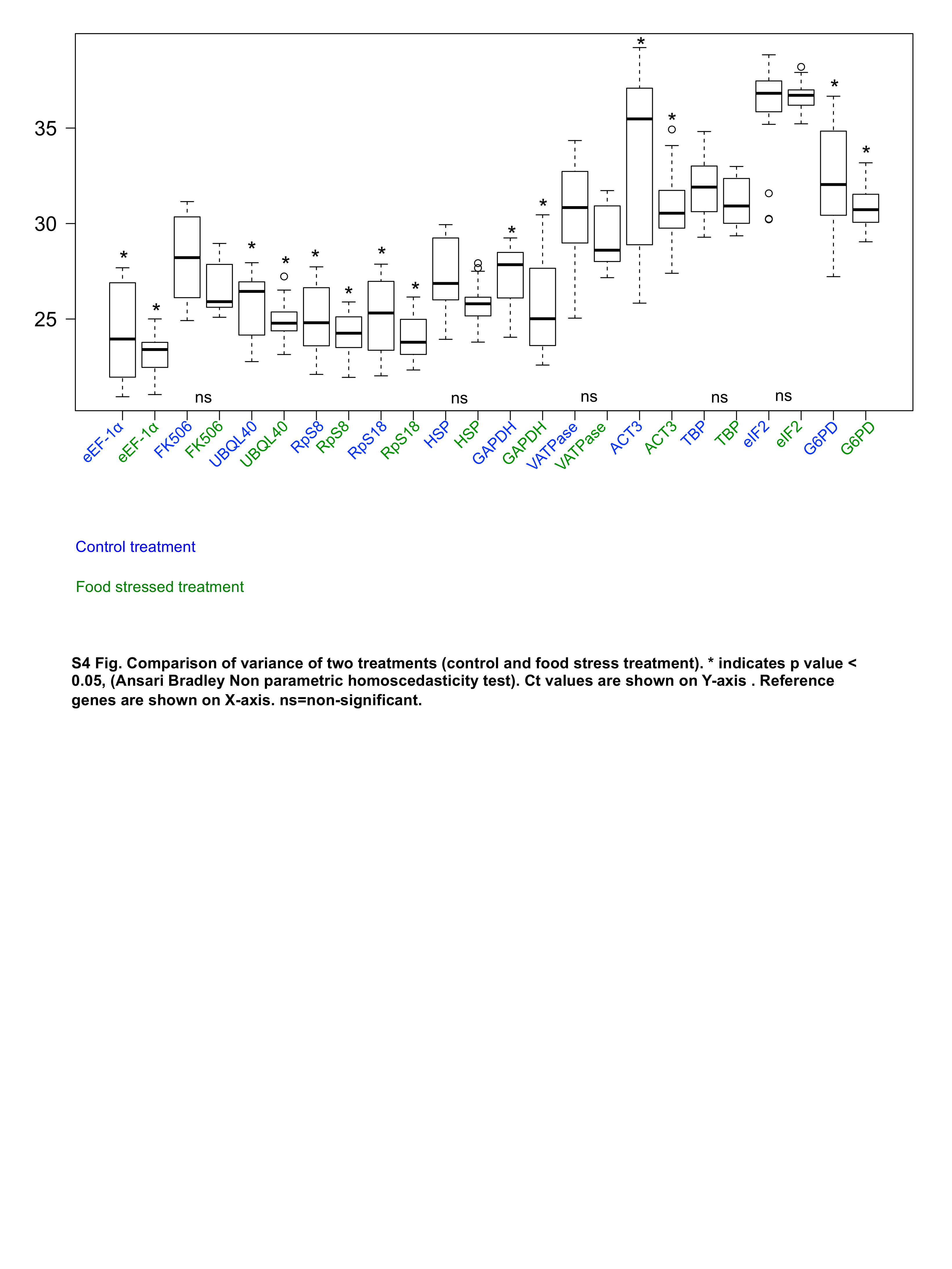

Supplement: S4 Fig — (TIF) [file pone.0120401.s004.tif]
